# Covid 19 pandemic unveiling the opportunities and challenges in orthodontic training

**DOI:** 10.1590/2177-6709.25.3.007-008.edt

**Published:** 2020

**Authors:** Flavia Artese

**Affiliations:** 1 Universidade do Estado do Rio de Janeiro, Departamento de Odontologia Preventiva e Comunitária (Rio de Janeiro/RJ, Brazil).

The COVID-19 pandemic disrupted every single aspect of society, with most professions having to rapidly adapt to the new circumstances, and these were affected at different levels due to specific demands of contamination control. In the USA, OSHA (Occupational Safety and Health Administration) early on classified Dentistry as a ‘very high-risk’ category, having doctors, staff and patients themselves exposed to aerosol-generating procedures. Clearly, this contamination risk not only impacted orthodontic practice, but also orthodontic training.

Guidelines for clinical activities in dentistry were established^1^ and, in general, measures had the purpose of avoiding transmission routes through droplets or direct contact. Changes in practice were drastic, and affected the entire clinical setting, from the front desk all the way to the surgery. And these guidelines obviously had to be carried over to clinical orthodontic training. 

Face to face education as a whole was halted, since social distancing was mandatory for reducing contamination. The conventional teaching model of a teacher and a classroom full of students was most probably the first thing to stop in most countries. E-learning became the main option, and many dental schools had to shift to this teaching model with very little or no practice at all in distance learning. Orthodontic education was no different, and very little information or recommended guidelines were available to ensure the fulfillment of its curriculum.

In collaborative efforts, specialty organizations, such as the Angle East and the Angle Society of Europe, put together 8 weeks’ worth of one-hour seminars, offered daily to orthodontic graduate students. Over 300 programs worldwide had free enrollment and their residents were exposed to a wealth of knowledge not attainable in different circumstances. Individual efforts were also evident, such as Kevin O’Brien’s paper discussions, to mention a few of the several others available on different electronic media.

Nevertheless, dental education faces serious challenges, as two recent papers point out these aspects in the United States,^2^ ^,^ ^3^ the data can be extrapolated to other countries. Most dental schools have to deal with its multiple functions in society, which are not limited to teaching, but also include patient treatment and research. This is not different in orthodontic graduate training, since orthodontics, as most dental specialties, is of an operatory nature and a key part of education is performed in the surgeries or clinics.

The Association for Dental Education in Europe (ADEE), with the aim of getting the picture of the first response of European dental schools to the COVID-19, sent out an electronic survey to 153 dental schools.^4^ The responses were collected from March 25^th^ to April 5^th^, 2020 and covered five main areas: clinical activities, non-clinical teaching, assessment, emotional support and future implications. Results showed that non-clinical teaching was performed online in 90% of the schools. Evaluations were postponed in 72% of the schools, since clinical requirements could not be performed. The well-being of staff and students were managed centrally by 50% of the schools, with dedicated web pages and online meetings. Finally, 90% of the schools believed that COVID-19 crisis will change permanently dental education.

The greatest challenge is that orthodontic education has a high need of clinical activities. Despite the technology advances that include robots and electronic typodonts, these are not portable resources and cannot be used to replace hands-on clinical training. Also, the patient-doctor relationship can only be learnt in a physical clinical setting. To be able to offer clinical training at this time, modifications in clinical settings will be needed, demanding large financial investments; and as clinical activity will be slower, involving less people, this will be the most significant obstacle in offering the orthodontic resident the fulfillment of his/her training with no loss in quality.

Research has never been so valued by society in searching for a vaccine or medication for this disease.^5^ However, it has also affected orthodontic research, especially those performed in a clinical setting. The research financing and the deadlines of research grants will probably not be extended, as well as the completion of these projects as orthodontic graduation requirements. Needless to say the impact on future research grants, as well as the financial impact on universities as a whole, since most of them are dependent on tuition fees.

Despite the enthusiasm with the fact that orthodontic residents have never been exposed to such amount of theoretical information during the lockdown period, and the value that e-learning almost instantly received due to the pandemic, some other challenges lie ahead of us. Educators involved in orthodontic training have to be very careful in respect to changes in teaching resources and dynamics. Even though some aspects of teaching can and will be adequately substituted by online lecturing, treatment skills are yet to be developed and performed on patients, which is not only about technique, but understanding of patient-doctor relationship. This pandemic has come for a definite change, but we have to be careful in offering substitutes to teaching, so as not to inflict on quality of learning. I believe a completely new demand of research on orthodontic education is ahead of us, so that these changes will also be based on evidence, rather than on the urgency of graduating our current students. 

May the excellence in quality of orthodontic training prevail.
